# Clinical characteristics and outcomes of the multisystem inflammatory syndrome in children (MIS-C) following COVID-19 infection in Iran: A multicenter study

**DOI:** 10.1371/journal.pone.0274104

**Published:** 2022-09-22

**Authors:** Fereshteh Rostami-Maskopaee, Fani Ladomenou, Seyedeh-Kiana Razavi-Amoli, Mohammad Reza Navaeifar, Azin Hajialibeig, Leila Shahbaznejad, Fatemeh Hosseinzadeh, Behzad Haghighi Aski, Ali Manafi Anari, Mohsen Mohammadi, Mohammad Bagher Rahmati, Eslam Shorafa, Seyedenarjes Abootalebi, Mohammad Sadegh Rezai

**Affiliations:** 1 Pediatric Infectious Diseases Research Center, Communicable Diseases Institute, Mazandaran University of Medical Sciences, Sari, Iran; 2 Venizeleion General Hospital, Heraklion, Crete, Greece; 3 Research Committee, School of Medicine, Mazandaran University of Medical Sciences, Sari, Iran; 4 Department of Pediatrics, Ali Asghar Children’s Hospital, Iran University of Medical Sciences, Tehran, Iran; 5 Non-Communicable Pediatric Diseases Research Center, Health Research Institute, Babol University of Medical Sciences, Babol, Iran; 6 Department of Pediatrics, School of Medicine, Bandar Abbas University of Medical Sciences, Bandar Abbas, Iran; 7 Division of Intensive Care Unit, Department of Pediatrics, School of Medicine, Shiraz University of Medical Sciences, Shiraz, Iran; Institut National de la Santeet de la Recherche Medicale (INSERM), FRANCE

## Abstract

**Objectives:**

This study aimed to assess the clinical characteristics, treatment and outcomes of the multisystem inflammatory syndrome in children (MIS-C) following COVID-19 in five different geographical regions of Iran.

**Methods:**

In this multicenter observational study, patients <21 years were included between March 2020 and October 2021. By Disease Control and Prevention (CDC) checklist, demographic characteristics, comorbidities, clinical signs and symptoms, laboratory and radiology findings, and treatment were collected. Statistical analysis was using Chi-square and t-test in STATA_14_.

**Results:**

In total 225 patients with median age of 55 (26–96) months were included that 59.56% boys. 57.33% were admitted to the PICU with a median of 7 days (4–10). 95.56% of patients were discharged with recovery and the rest died. All of the patients in our study were included based on the MIS-C criteria. However, some patients had Kawasaki symptoms, so we compared the clinical and epidemiological characteristics of the two groups. Conjunctival injection, cervical lymphadenopathy>1.5 cm diameter, and strawberry tongue in Kawasaki-like MIS-C patients were higher than of MIS-C patients, and this difference was significant(p<0.001). The most common comorbidity was obesity (24.86%). Most patients tested for COVID-19 and about 60% of the patients had a positive test by serology or reverse transcription-polymerase chain reaction (RT-PCR). Gastrointestinal (88.89%) and hematologic signs (84.44%) were most common. Most drugs used in patients were IVIG and steroids. 88.07% and 61.29% of the patients had at least one problem in echocardiography and lung CT, respectively.

**Conclusions:**

The best outcome was seen in patients who were treated with both IVIG and steroids on the first days of admission. Myocarditis was common in two groups of patients. According to most patients had echocardiography abnormal, screening of heart function is recommended for patients.

## Introduction

Worldwide, the urgent dissemination of a novel entity termed severe acute respiratory syndrome coronavirus 2 (SARS‐CoV‐2) has been stated as one of the most challenging health issues [1]. Initially, coronavirus disease 2019 (COVID-19) exhibited clinically various presentations ranging from asymptomatic manifestation to multiple organ dysfunction in critically ill patients [2]. In the spring of 2020, an unexpected phenomenon illustrating a hyperinflammatory syndrome with multi-organ involvement has been identified in clusters of children, possibly associated with COVID-19 [3, 4]. Afterward, as similar cases subsequently appeared globally, the Centers for Disease Control and Prevention (CDC) and World Health Organization (WHO) reached a consensus to announce this potentially life-threatening circumstance during COVID-19 as a multisystem inflammatory syndrome in children (MIS-C) [5]. As of October 2021, there have been approximately 5,217 cases of MIS-C, including 46 deaths reported in the United States [6]. Diagnostic criteria for MIS-C in critically ill patients necessitate fever, elevated inflammatory markers, and involvement of at least two organs, with evidence of COVID-19 infection or exposure within four weeks before symptoms onset, and exclusion of other obvious etiologies [7]. MIS-C may have similarities with Kawasaki Disease (KD), including mucocutaneous involvement, conjunctivitis, lymphadenopathy, and elevation of inflammatory markers. Since the emergence of MIS-C, debates regarding distinguishing between MIS-C and KD are ongoing among specialists globally [8].

Nevertheless, some distinct clinical features correlated with MIS-C make a considerable difference from KD, such as prominent gastrointestinal symptoms, lymphocytopenia, and prevalent cardiovascular implications that demanded the urgent multidisciplinary management in MIS-C [9]. The exact underlying pathological mechanism of MIS-C is not still elucidated.

Regarding the COVID-19 variants that continue to emerge, the severity and outcomes of MIS-C can be significantly affected. Geographic, genomic, and ethnic distributions of children with MIS-C need to be considered to identify the exact diagnostic criteria and optimal treatment guidelines. Further comprehensive studies are required to improve the insights into MIS-C manifestations, treatments, outcomes, and prevention in children. Hence, we performed this multicenter observational study to assess the spectrum of clinical characteristics and outcomes of MIS-C in children following COVID-19 in five referral hospitals in Iran during 2020–2021.

## Materials and methods

The multicenter observational study included children <21 years who fulfilled the MIS-C criteria according to the CDC checklist [10]. The patients were hospitalized between March 2020 and September 2021 in five referral hospitals in Iran; including Ali Asghar children’s hospital of Tehran, Namazi hospital of Shiraz, Booali Sina hospital of Sari, Children’s hospital of Bandar Abbas, and Shafizadeh hospital of Babol. Patients with MIS-C were identified at each center by subspecialty physicians. Positive tests for current SARS-CoV-2 infection or exposure to COVID-19 case within four weeks prior to symptoms onset are a principal component of the CDC criteria for MIS-C. With regards to that, a nasopharyngeal reverse transcription-polymerase chain reaction (RT-PCR) assay was performed for most of the suspected SARS-CoV-2 patients. Furthermore, an enzyme-linked immunosorbent assay (ELISA) was done for identifying serum levels of anti-SARS-CoV-2 immunoglobulin G and M (Pishtaz Teb Kit). Since the beginning of the MIS-C emergence, adequate information about MIS-C was lacking, cases associated with multi-organ involvement manifestations with an epidemiological link to COVID-19 were considered suspected and included in our study without the requirement of a positive COVID-19 test.

### Data collection and analysis

Medical records were extracted from admission to discharge by skilled staff onto a checklist. Data concerning patient demographic characteristics, comorbidities, clinical signs and symptoms on admission and during hospitalization, laboratory assay results, radiology findings (Echocardiogram, Abdominal and Chest imaging), complications, diagnostic tests (SARS-CoV-2 PCR and serology tests), treatments and need for pediatric intensive care unit (PICU) admission were recorded.

Based on the CDC case definition, the following criteria were considered to define MIS-C patients: 1) age<21 years; 2) fever≥ 38.0°C (100,4°F) for >24 hours or report of subjective fever lasting> 24 hours; 3) a history of current SARS-CoV-2 infection by positive RT-PCR, serology, or antigen test or contact with a suspected or confirmed COVID-19 case within 4 weeks prior to the onset of symptoms; 4) signs and symptoms of multisystem (≥2) organ involvement including: cardiovascular (e.g. shock, elevated troponin), renal, gastrointestinal (e.g. abdominal pain, vomiting), hematologic (e.g. coagulopathy), neurologic (e.g. seizure, meningitis) and respiratory (e.g. pneumonia, pulmonary embolism); 5) positive inflammatory markers including elevated CRP, erythrocyte sedimentation rate (ESR), D-dimers, ferritin, interleukin 6 (IL-6), elevated neutrophils, lymphocytopenia, low albumin; 6) no apparent plausible diagnosis [10].

According to the CDC checklist, if the patient has one of the results of coronary artery aneurysms, including coronary artery dilation, cardiac dysfunction, mitral regulation (MR), tricuspid regulation (TR), pulmonary valve insufficiency, pericardial dysfunction, and low ejection fraction (EF) < 55%, were considered as abnormal results in echocardiography. Abdominal sonography was considered abnormal if the patient had one of the following criteria: free fluid, mesenteric lymphadenopathy or splenomegaly. Moreover, chest CT scans were reported as abnormal if one of the following were present: Ground-glass Opacity (GGO), consolidation, pneumonia, pleural effusion, or atelectasis.

Based on the WHO child growth standards, obesity was considered as the body mass index(BMI) at or over the 95th percentile for age and sex [9]. BMI was calculated as weight in kilograms divided by height in meters squared.

### Ethics statement

This study was approved by the ethics committee of Mazandaran University of Medical Sciences (IR.MAZUMS.REC.1400.10565). Written consent was obtained from the parents or guardians of all patients. In addition, we got verbal consent from the 10 years and older patients.

Results were represented as mean ± standard deviation (SD) or median with interquartile range (IQR) for continuous variables and frequency with percentage for categorical variables. All of the patients met the MIS-C criteria; however, some patients had Kawasaki symptoms. Based on clinical manifestations and laboratory markers, the patients were divided into two groups: Kawasaki-like MIS-C and MIS-C. Kawasaki-like MIS-C is a child who meets the criteria for complete or incomplete KD with positive RT-PCR or increased IgM, IgG COVID-19.

Comparisons were assessed using the Chi-square and t-test (Mann-Whitney test) based on the data distribution. Statistical analyses were conducted using STATA_14_. A p-value less than 0.05 was considered significant.

## Results

### Study population and characteristics

A total of 225 patients were included in the study; 59.6% boys and 40.4% girls. The sex ratio of patients was 1.47 (134 males, 91 females). The median age of all patients was 55 months (26–96). Patients were included from Shafizadeh Children’s hospital (30.7%), Booali Sina hospital (27.6%), Ali Asghar hospital (17.8%), Namazi hospital (12.9%), and Bandar Abbas Children’s hospital (11.1%), respectively. 129/225 (57.3%) patients were admitted to the PICU with a median length of stay of seven days (4–10). Two hundred fifteen patients (95.6%) were discharged from the hospital with complete or partial recovery, and ten patients (4.44%) died (Table 1).

**Table 1:** The baseline characteristics and clinical outcome in MIS-C and Kawasaki- like MIS-C.

Characteristic	Total(N = 225)	Kawasaki- like MIS-C	MIS-C	p-value
		(n = 58)	(n = 167)	
**Age in month, median (IQR)**	55(26–96)	56(32–89)	54(24–96)	0.94
**Gender, n (%)**				
Male	134(59.6)	38(65.5)	96(57.5)	0.28
Female	91(40.4)	20(34.5)	71(42.5)	
**Comorbid** ^**a**^				
Yes	73(32.4)	15(25.9)	58(34.7)	0.21
No	152(67.6)	43(74.1)	109(65.3)	
**Type of comorbid** ^**a**^				
Obesity ^b^	44(24.9)	7(14.3)	37(28.9)	**0.04**
Seizures	17(7.6)	4(6.90)	13(7.78)	0.82
Chronic lung disease	12(5.33)	5(8.62)	7(4.19)	0.19
Immunosuppressive disorder malignancy	3(1.33)	0	3(1.80)	0.57
Type1 diabetes	3(1.33)	0	3(1.80)	0.57
G6PD insufficient	16(7.11)	5(8.62)	11(6.59)	0.60
**Hospital stay(days), median (IQR)**	9(7–13)	9(7–12)	9(7–13)	0.82
**PICU admission**	129(57.3)	33(56.9)	96(57.5)	0.93
**ICU stay(days), median (IQR)**	7(4–10)	8(6–11)	7(4–9.5)	0.05
**Outcome**				
Survived	215(95·6)	58(100)	157(94.0)	0.06
Died	10(4·44)	0	10(5.99)	

^a^ A patient may have at least one type of comorbid.

^b^ The number of available patient height and weight information for calculating body mass was 177 patients.

Based on their clinical manifestations and laboratory markers, the patients were divided into two categories: Kawasaki-like MIS-C (n = 58) and MIS-C (n = 167).

The mean age of patients in MIS-C group was higher than Kawasaki-like MIS-C patients, [64 ± 48 months (range 2–180) compared to 59 ± 34 months (range 9–125)]. Regarding the comorbid variable, 34.7% of MIS-C patients had at least one type of comorbid disease compared to 25.9% of Kawasaki-like MIS-C. Obesity was the most common comorbid in both groups. This difference was statistically significant (p 0.044).

The median number of hospital stay in both groups was nine days. The median number of ICU hospitalization days was eight days in Kawasaki like MIS-C and seven days in MIS-C patients. This difference was marginally significant (p 0.059).

### Clinical signs and symptoms

All patients had fever at the time of admission. The median duration of fever was eight days (6–10) with statistically significant difference between the two groups of patients (p 0.002). The most common signs and symptoms were gastrointestinal 88.9%, hematologic 84.4% and skin 61.3% of the patents. There was a significant difference between cardiac and skin symptoms, conjunctival injection, cervical lymphadenopathy, and strawberry tongue between the two groups of patients (p <0.05) (Table 2).

**Table 2:** Clinical signs and symptoms in Kawasaki- like MIS-C and MIS-C patients.

Clinical Signs and Symptoms	Total(N = 225)	Kawasaki- like MIS-C	MIS-C	p-value
		(n = 58)	(n = 167)	
**Fever**	225(100)	58(100)	167(100)	
**Duration of fever(days), median (IQR)**	8(6–10)	8(7–10)	7(5–10)	**0.001**
**Cardiac**				
Yes	109(48.4)	20(34.5)	89(53.3)	**0.01**
No	116(51.6)	38(65.5)	78(46.7)	
**Renal**				
Yes	83(36.9)	24(41.4)	59(35.3)	0.41
No	142(63.1)	34(58.6)	108(64.7)	
**Respiratory**				
Yes	121(53.8)	32(55.2)	89(53.3)	0.80
No	104(46.2)	26(44.4)	78(46.7)	
**Hematologic**				
Yes	190(84.4)	48(82.7)	142(85.0)	0.68
No	35(15.6)	10(17.2)	25(14.9)	
**Gastrointestinal**				
Yes	200(88.9)	53(91.4)	147(88.0)	0.48
No	25(11.1)	5(8.6)	20(11.9)	
**Skin**				
Yes	138(61.3)	53(91.4)	85(50.9)	**<0.001**
No	87(38.7)	5(8.62)	82(49.1)	
**Neurologic**				
Yes	63(28)	15(25.9)	48(28.7)	0.67
No	162(72)	43(74.1)	119(71.3)	
Other				
**Conjunctival injection**	88(39.1)	46(79.3)	42(25.2)	**<0.001**
**Cervical lymphadenopathy >1.5 cm diameter**	21(9.33)	13(22.4)	8(4.8)	**<0.001**
**Strawberry tongue**	16(7.11)	15(25.9)	1(0.60)	**<0.001**

### Complications

The most common complications were myocarditis in 35.1%, hypotension in 26.7%, and shock in 21.8%. There was a significant difference in hypotension, shock, arrhythmia, pneumonia, and congestive heart failure in Kawasaki- like MIS-C and MIS-C patients (p <0.05) (Table 3).

**Table 3:** Complication in Kawasaki- like MIS-c and MIS-c patients.

Complication	Total(N = 225)	Kawasaki- like MIS-C	MIS-C	p-value
		(n = 58)	(n = 167)	
Myocarditis	79(35.1)	22(37.9)	57(34.1)	0.60
Hypotension	60(26.7)	5(8.62)	55(32.9)	**<0.001**
Shock	49(21.8)	3(5.17)	46(27.5)	**<0.001**
Arrhythmia	45(20)	19(32.7)	26(15.6)	**0.005**
Pneumonia	45(20)	4(6.90)	41(24.5)	**0.004**
Congestive heart failure	21(9.33)	0	21(12.6)	**0.003**
ARDS^a^	7(3.11)	0	7(4.19)	0.19
Pericarditis	6(2.67)	0	6(3.59)	0.34

^a^ Acute respiratory distress syndrome.

### Treatment

The most commonly prescribed medication were IVIG, steroids, antiplatelet and anticoagulant, with 94.2%, 77.8%, 68.4% and 54.7%, respectively. There was a statistically significant difference between the drugs used in the two groups of patients, including steroids, antiplatelet, antibiotic, antiviral, second IVIG, and high flow nasal cannula (P <0.05). Among the drugs steroids and IVIG were marginally significant in two groups of patients (p 0.054) (Table 4).

**Table 4:** Treatment in Kawasaki- like MIS-C and MIS-C patients.

Treatment	Total(N = 225)	Kawasaki- like MIS-C	MIS-C	p-value
		(n = 58)	(n = 167)	
IVIG	211(94.2)	57(98.3)	154(92.8)	0.12
Steroids	175(77.8)	37(63.8)	138(82.6)	**0.003**
Steroids and IVIG	161(71.9)	36(62.1)	125(75.3)	0.05
Antiplatelet	154(68.4)	49(84.5)	105(62.8)	**0.002**
Anticoagulation	123(54.7)	27(46.5)	96(57.5)	0.15
Antibiotics	123(54.7)	50(86.2)	73(43.7)	**<0.001**
Vasoactive medication	100(44.4)	26(44.8)	74(44.3)	0.94
Low flow nasal cannula	90(40)	21(36.2)	69(41.3)	0.49
Second IVIG	38(16.9)	4(6.90)	34(20.4)	**0.01**
Antivirals	38(16.9)	20(34.5)	18(10.8)	**<0.001**
Intubation	22(9.78)	2(3.45)	20(11.9)	0.06
Mechanical ventilation	21(9.33)	2(3.45)	19(11.4)	0.07
High flow nasal cannula	13(5.78)	0	13(7.78)	**0.02**
Immune modulators	12(5.33)	1(1.72)	11(6.59)	0.15
Noninvasive ventilation	5(2.22)	0	5(2.99)	0.33
Dialysis	2(0.89)	0	2(1.20)	1

### Imaging

Echocardiography was performed in 218/225 patients (96.9%), and at least one problem was detected in 88.1%. The most common complications were MR (88.7%), TR (64.1%), and cardiac dysfunction (51.6%), respectively. One hundred nine patients had both MR and TR. There was a statistically significant difference in TR, coronary artery dilatation, pulmonary valve insufficiency and low EF in these two groups (p <0.05), being more commonly in the MIS-C group. In this study, 146 patients (64.9%) underwent an abdominal ultrasound, and 54.1% of these patients had problems and the most common was free fluid (76.3%). A significant difference was in the presence of mesenteric lymphadenopathy between these two groups (p<0.05). Only 155 patients (68.9%) had spiral chest CT scans, and 61.3% of them had abnormal findings, the most commonly being ground-glass opacity and consolidation in 84.2% (Table 5).

**Table 5:** Characteristic of imaging in Kawasaki- like MIS-C and MIS-C patients.

	Total(N = 225)	Kawasaki- like MIS-C	MIS-C	p-value
		(n = 58)	(n = 167)	
**Echocardiography** ^**a**^				
Yes	192(88.1)	52(91.2)	140(86.9)	0·39
No	26(11.9)	5(8.77)	21(13.0)	
Mitral Regulation	157(81.8)	45(86.5)	112(80)	0.40
Tricuspid regurgitation	123(64.1)	41(78.9)	82(58.6)	**0.009**
Cardiac dysfunction	99(51.6)	24(46.2)	75(53.6)	0.31
Ventricular dysfunction	93(48.4)	22(42.3)	71(50.7)	0.30
Coronary artery dilatation	62(32.3)	25(48.1)	37(26.4)	**0.004**
Pericardial Effusion	60(31.3)	20(38.5)	40(28.6)	0.18
Brightness & PVB	54(28.1)	19(36.5)	35(25)	0.11
Lack of tapering	54(28.1)	22(42.3)	32(22.9)	**0.008**
Low EF	51(26.6)	21(40.4)	30(21.4)	**0.008**
Pulmonary valve insufficiency	36(18.8)	19(36.6)	17(12.1)	**<0.001**
Coronary artery aneurysms	2(1.04)	1(1.92)	1(0.71)	0.46
**Abdominal Sonography** ^**a**^				
Yes	79(54.1)	24(63.2)	55(50.9)	0.19
No	67(45.9)	14(36.2)	53(49.1)	
Free fluid	61(76.3)	21(84)	40(72.7)	0.27
Mesenteric lymphadenopathy	34(42.5)	6(25)	28(50)	**0.03**
Splenomegaly	6(7.50)	1(4.17)	5(8.93)	0.66
**Spiral Chest CT scan** ^**a**^				
Yes	95(61.3)	21(70)	74(59.2)	0.27
No	60(38.7)	9(30)	51(40·8)	
Ground*-*glass Opacity (GGO) and Consolidation	80(84.2)	19(90.5)	61(82.4)	0.37
Pleural Effusion	40(42.1)	10(47.6)	30(40.5)	0.56
Atelectasis	21(22.3)	2(9.52)	19(26.0)	0.11

^a^ A patient may have at least one type of abnormal results so; the total percentage is greater than 100.

Most patients had been tested for COVID-19, and 60% had a positive test by serology or RT-PCR. Moreover, 76.8% of patients had exposure to confirmed COVID-19 cases. Thirteen patients (6.70%) had positive both RT-PCR and serology for SARS-CoV-2. Leukopenia, ESR >30, elevated troponin and D-dimer >1000 were statistically significant in two group of patients (P<0.05). NT Pro-BNP ≥110 in Kawasaki-like MIS-C and MIS-C patients was marginally significant (p 0.059). In this study, NT pro-BNP, troponin, IL6, fibrinogen and ferritin were only evaluated in some patients (Table 6).

**Table 6:** Laboratory features in Kawasaki- like MIS-C and MIS-C patients.

laboratory Features	N (%)	Kawasaki- like MIS-C (n = 58)	MIS-C (n = 167)	P-value
**COVID-19 Test Results**				
Yes	116(59.8)	26(50.9)	90(62.9)	0.13
No	78(40.2)	25(49.0)	53(37.0)	
**Positive RT-PCR**	49(25.3)	14(27.5)	35(24.5)	0.67
**Positive Serology**	79(40.7)	20(39.2)	59(41.3)	0.79
**Positive RT-PCR & Serology**	13(6.70)	8(15.7)	5(3.50)	**0.003**
**Exposure to COVID-19**	172(76.8)	46(79.3)	126(75.9)	0.59
**WBC<4000**^**a**^ **(highest) (n = 220)**	8(3.64)	3(5.26)	5(3.07)	0.44
**WBC<4000**^**a**^ **(lowest) (n = 184)**	40(21.7)	5(10.6)	35(25.6)	**0.03**
**WBC > = 10000**^**b**^ **(highest) (n = 220)**	158(71.8)	44(77.2)	114(69.9)	0.29
**WBC > = 10000**^**b**^ **(lowest) (n = 184)**	28(15.2)	7(14.9)	21(15.3)	0.94
**PLT<100000**^**c**^ **(highest) (n = 221)**	7(3.17)	0	7(4.27)	0.19
**PLT<100000**^**c**^ **(lowest) (n = 187)**	64(34.2)	13(27.1)	51(36.7)	0.22
**PLT> = 450000**^**d**^ **(highest) (n = 221)**	81(36.6)	23(40.4)	58(35.4)	0.50
**PLT> = 450000**^**d**^ **(lowest) (n = 187)**	17(9.09)	5(10.4)	12(8.63)	0.71
**ESR>30 (highest) (n = 135)**	122(90.4)	51(98.1)	71(85.5)	**0.01**
**ESR>30 (lowest) (n = 73)**	42(57.5)	14(46.7)	28(65.1)	0.11
**ESR>100 (highest) (n = 135)**	29(21.5)	8(15.4)	21(25.3)	0.17
**ESR>100 (lowest) (n = 73)**	4(5.48)	0	4(9.30)	0.13
**D-Dimer >1000 (n = 169)**	120(71.0)	34(87.9)	86(66.2)	**0.01**
**CRP (mg/L)> = 10 (n = 222)**	205(92.3)	56(96.6)	149(90.9)	0.16
**Ferritin <12 & >135(n = 123)**	104(84.6)	22(88)	82(83.7)	0.59
**Fibrinogen<200 & >400(n = 63)**	37(58.7)	2(28.6)	35(62.5)	0.08
**NT pro BNP> = 110(n = 167)**	97(58.1)	37(68.5)	60(53.1)	0.05
**Troponin> = 0.3(n = 136)**	52(38.2)	4(14.3)	48(44.4)	**0.003**
**IL6> = 5.9(n = 18)**	16(88.9)	2(100)	14(87.5)	1

^a^ leukopenia

^b^ Leukocytosis

^c^ Thrombocytopenia

^d^ Thrombocytosis.

## Discussion

Since the appearance of MIS-C, physicians have been encountering challenges in diverse manifestations of MIS-C that could lead to misdiagnosis. In this observational study, we described the clinical, laboratory and demographic characteristics of MIS-C and Kawasaki like MIS-C in 225 children and adolescents <21 years in Iran. Although, MIS-C and KD share similarities through clinical presentations, laboratory findings, and treatment; there are particular distinctions between MIS-C and KD that potentially facilitates prompt diagnosis and multidisciplinary management in critically ill patients.

In our study, the median age of the patients with MIS-C and Kawasaki like MIS-C was 4.5 and 4.6 years, respectively. However, some reports have shown that MIS-C tends to affect all age groups, especially children older than five years, with a median age ranging from 6 to 12 years [11, 12].

Consistently with other studies [13–15], a male predominance was detected among our MIS-C patients. Biological dissimilarities between males and females may impact the immune reaction to SARS-CoV-2, as has been discussed in KD [16]. However, the effect of gender on the prevalence, severity and mortality of MIS-C needs to be clarified by larger studies.

Initially, it was presumed that as most patients diagnosed with MIS-C were previously healthy, underlying comorbid diseases were not substantially associated with MIS-C. Nevertheless, in line with study by Abram et al. in our study, obesity was the most prevalent comorbidity, counting for 24.9% of the MIS-C patients [17].

In the current study, the median duration of hospital stay was similar in both MIS-C and Kawasaki-like MIS-C groups, with a median duration of 9 (7–13) days. In the literature, the median length of hospital stay in MIS-C patients ranged from 6 to 13 days [18–20]. It is important to notice that initial treatment can impact the average duration of hospitalization. In our study, most patients received IVIG and steroids as the main treatments and it is in agreement with other studies [14, 21]. Regarding the more utilization of steroids plus IVIG in the MIS-C group, we found that the median duration of PICU stay was shorter in these patients. About 17% of the patients needed a second dose of IVIG due to persistent major signs and symptoms. These patients had a fever for more than five days. In children with Kawasaki-like MIS-C, there was a clinical reassessment 36 hours after IVIG infusion and if they did not respond to IVIG treatment, they received a second dose of IVIG.

In a systematic review by Kaushik et al. MIS-C was associated with favorable outcomes in most of patients and 11 (1.7%) MIS-C related deaths were observed among 655 patients [14]. However, we observed that the mortality ratio in our patients was 4.8%. Importantly, diverse factors including age, ethnicity, gender, prompt diagnosis, and accurate treatment may potentially impact the prognosis of MIS-C [22, 23].

With regards to the symptoms, fever is encountered as the most common characteristic in MIS-C and KD patients [24]. We noticed that duration of fever was longer in Kawasaki-like MIS-C, compared to the MIS-C group. Likewise, Bar-Meir et al. reported the same duration of fever in patients who met both MIS-C and KD criteria’s [11].

Our observations about clinical manifestations of MIS-C were in agreement with those in the literature [14, 25]. However, respiratory and renal involvement was more common in our study. Cardiac involvement was reported as a more frequent feature in patients with MIS-C compared to the Kawasaki-like MIS-C group. Specially, we found that classic presentations of KD, including the strawberry tongue, lymphadenopathy, and conjunctivitis, were less frequent in the MIS-C group compared to the Kawasaki-like MIS-C group.

In our study, 88% of the patients had abnormal echocardiographic results, which were higher in the Kawasaki-like MIS-C patients and MR was the most common problem. In the Blumfield et al. study, 81% of patients had abnormal echocardiographic results that were almost similar to our study [26]. In a Mamishi et al. study, 56% of the patients had cardiac involvement [21]. It should be noted that the reasons for the difference in results can be operator-dependent. Also, according to the hospital policy, not all patients may have an echocardiogram and only seriously ill patients underwent it. On lung CT scan, 61.3% of the patients had one of the problems including GGO or consolidation, pleural effusion, or atelectasis. In the Kwak et al. study, 13 to 41% of patients reported opacity and infiltration, while in our study, GGO and consolidation were commonly reported (84.21%) [27].

In agreement with some studies, most of our patients had positive COVID-19 tests or contact with a COVID-19 positive person [21, 27].

Our findings of high inflammatory markers, including ESR and CRP at the time of admission, were similar to those in other studies [14, 27].

In the current study, D-dimers over 1000 were reported in 71% of the patients and there was a significant difference between the two groups. Also, seven out of 10 patients who died had D-dimer over 1000. In several studies D-dimer has been demonstrated as a biomarker for disease severity and mortality in COVID-19 patients [28, 29]. Ferritin was abnormal in 84.5% of our patients. Ina study, abnormal ferritin was found in 73% [21]. Ferritin is not evaluated routinely for all patients because its role in monitoring inflammation is still not very clear. In a systematic review and meta-analysis, most patients had leukopenia with marked Lymphopenia [30]. In our study, leukopenia was higher in MIS-C patients, and this difference was statistically significant. In another study, patients had leukopenia but did not see a significant difference in leukopenia at the time of admission in patients [31].

We had some limitations that should be noted. First, data collection in some hospitals was from the medical records of hospitalized patients, so there was no access to information and variables that needed to be checked in other hospitals. Second, imaging was not performed on all patients, or we did not have an imaging document of patients to be evaluated by two separate radiologists for agreement percentage.

## Conclusion

All of the patients in our study met the MIS-C criteria; however some patients had Kawasaki symptoms, so we compared the clinical and epidemiological characteristics of the two groups. Myocarditis was common in two groups of patients. Conjunctival injection, cervical lymphadenopathy>1.5 cm diameter, and strawberry tongue in Kawasaki-like MIS-C patients were higher than of MIS-C patients. The most common symptom in patients was gastrointestinal symptoms. The best outcome was seen in patients who were treated with both IVIG and steroids on the first days of admission. According to most patients who had echocardiography abnormal, screening of heart function is recommended for patients.
