# Correction: Clinical characteristics and outcomes of the multisystem inflammatory syndrome in children (MIS-C) following COVID-19 infection in Iran: A multicenter study

**DOI:** 10.1371/journal.pone.0314626

**Published:** 2024-11-21

**Authors:** Fereshteh Rostami-Maskopaee, Fani Ladomenou, Seyedeh-Kiana Razavi-Amoli, Mohammad Reza Navaeifar, Azin Hajialibeig, Leila Shahbaznejad, Fatemeh Hosseinzadeh, Behzad Haghighi Aski, Ali Manafi Anari, Mohsen Mohammadi, Mohammad Bagher Rahmati, Eslam Shorafa, Seyedenarjes Abootalebi, Mohammad Sadegh Rezai

There is an error in affiliation 1 for author Fereshteh Rostami-Maskopaee. The correct affiliation 1 is: Pediatric Infectious Diseases Research Center, Communicable Diseases Institute, Student Research Committee, Mazandaran University of Medical Sciences, Sari, Iran.
